# After Gestational Diabetes: Impact of Pregnancy Interval on Recurrence and Type 2 Diabetes

**DOI:** 10.1089/biores.2018.0043

**Published:** 2019-03-25

**Authors:** Judith Bernstein, Aviva Lee-Parritz, Emily Quinn, Omid Ameli, Myrita Craig, Timothy Heeren, Ronald Iverson, Brian Jack, Lois McCloskey

**Affiliations:** ^1^ Department of Community Health Sciences, Boston University School of Public Health, Boston, Massachusetts.; ^2^ Department of Obstetrics and Gynecology, Boston University School of Medicine, Boston, Massachusetts.; ^3^ Biostatistics and Epidemiology Coordinating Center, Boston University School of Public Health, Boston, Massachusetts.; ^4^ Department of Health Law, Policy, and Management, Boston University School of Public Health, Boston, Massachusetts.; ^5^ OptumLabs, Boston, Massachusetts.; ^6^ Department of Biostatistics, Boston University School of Public Health, Boston, Massachusetts.; ^7^ Department of Family Medicine, Boston University School of Medicine, Boston, Massachusetts.

**Keywords:** gestational diabetes, pregnancy interval, type 2 diabetes onset

## Abstract

The contribution of pregnancy interval after gestational diabetes (GDM) to type 2 diabetes (T2DM) onset is a poorly understood but potentially modifiable factor for T2DM prevention. The purpose of this study was to assess the impact of GDM recurrence and/or delivery interval on follow-up care and T2DM onset in a sample of continuously insured women with a term livebirth within 3 years of a GDM-affected delivery. This is a secondary analysis of a cohort of 12,622 women with GDM, 2006–2012, drawn from a national administrative data system (OptumLabs Data Warehouse). We followed 1091 women with GDM who had a subsequent delivery within 3 years of their index delivery. GDM recurred in 49.3% of subsequent pregnancies regardless of the interval to the next conception. Recurrence tripled the odds of early T2DM onset within 3 years of the second delivery. Women with GDM recurrence had greater likelihood of glucose testing in that 3-year interval, but not transition to primary care for continued monitoring, as required by both American Congress of Obstetricians and Gynecologists (ACOG) and the American Diabetes Association (ADA) guidelines. In multivariable analysis, we found a trend toward increased likelihood of T2DM onset for short interpregnancy intervals (≤1 year vs. 3 year, 0.08). Pregnancy interval may play a previously unrecognized role in progression to T2DM. T2DM onset after GDM can be prevented or mitigated, but many women in even this insured sample did not receive recommended follow-up monitoring and preventive care, even after a GDM recurrence. The postpartum visit may be an ideal time to inform patients about T2DM prevention opportunities, and discuss potential benefits of optimal spacing of future pregnancies.

## Introduction

Gestational diabetes (GDM) carries a high risk of early onset type 2 diabetes (T2DM), with rates of up to 60% diagnosed in the first decade after a GDM delivery,^1^ and increases long-term cardiovascular risk.^2^ The purpose of this study was to assess the impact of interval between deliveries on recurrence of GDM and early T2DM onset in a sample of continuously insured women with a history of GDM in an index pregnancy. Previous investigations have been limited to small samples that followed women longitudinally (*n* = 32–344) and larger samples that analyzed prevalence in subsequent pregnancies after a GDM-affected delivery but did not follow individual women over time (*n* = 1266 in a population study from Massachusetts, and *n* = 3689 in an Australian study).^3–5^ Big data from OptumLabs Data Warehouse (OLDW) provided a cohort of continuously insured women with few financial barriers to care during pregnancy; this allowed us to isolate a sample of women with documented GDM, exclude those with pre-existing diabetes, and, because of data continuity, follow them through a second pregnancy and 3 years beyond that delivery to identify GDM recurrence and subsequent T2DM onset.

## Methods

### Sample

This study is a secondary inquiry from a larger investigation of predictors of follow-up after GDM,^6^ using national data from OLDW, a comprehensive, longitudinal, deidentified data warehouse derived from insurance claims, survey results, enrollment files, and other public and private data. Because rates of completeness for electronic health record data vary by measure, we did not include body mass index in this analysis, but we were able to draw from all other components of the dataset. The diagnosis of GDM was characterized conservatively, using at least two International Classification of Diseases, 9th revision (ICD-9) claims of 648.8 for last trimester outpatient care or one claim for inpatient care, with no previous claims data depicting visits for T2DM or T2DM-related therapies. The details of sample selection, determination of diagnosis, and specification-independent variables are available from the original publication of the parent study,^6^ in print and in online supplementary material.

### Inclusion criteria

Briefly summarizing the original sample selection procedures, we first identified all unique women with delivery of a first livebirth in the data set between January 1, 2006 and September 30, 2012, as represented by single or multiple claims for pregnancy, delivery and/or postpartum care, using the ICD-9. We then characterized the first GDM-affected livebirth in the system (ICD-9 GDM code 648.8x).

### Exclusion criteria

We excluded: (1) women who had an ICD-9 code or history of standard hypoglycemic therapy suggestive of pre-existing T2DM, either before the 28th week of the index pregnancy or immediately postpartum; (2) women who did not have continuous insurance coverage from 1 year prepregnancy to 3 years postdelivery, because our unit of analysis was adequacy of follow-up after GDM in women who had limited financial barriers to seeking care, and (3) women who lacked comprehensive validated demographic data or information about service providers and delivery institutions, because demographic and institutional factors have been shown to affect adequacy of follow-up care.

These exclusions, documented in a Strobe Diagram, figure 1 in the parent study,^6^ were large but necessary to achieve analytic goals. We assessed generalizability of the reduced sample through comparisons of included and excluded women, and proceeded with analyses only after comparability was determined. Included and excluded samples were similar on all key variables listed in Table 1. Findings from these comparability analyses are summarized in the Results section, and available in online supplementary material associated with the original publication.^6^

**Table 1.** Characteristics of Women with a Gestational Diabetes-Affected Pregnancy and Delivery Who Conceived a Second Livebirth Within a 3-Year Period Following an Index Case of Gestational Diabetes

Characteristic	*N* = 1091	%
Age		
35+	824	75.5
≤35	267	24.5
Race		
Asian	123	11.3
Black	64	5.9
Hispanic	123	11.3
White	781	71.6
Education		
High school graduate or less	320	29.3
Some college or degree	771	70.7
Excess gestational weight gain by the time of the index delivery	39	3.6
GDM therapy: medication during the third trimester preceding the index delivery	179	16.4
Time to conception after the index delivery (years)		
<1	331	30.3
1–2	459	42.1
>2	301	27.6
GDM recurrence in subsequent pregnancy	538	49.3

GDM, gestational diabetes.

The sample for the present study consists of women in the original sample with a GDM-affected index delivery from January 2006 through September 2012 who had a subsequent delivery within 3 years after the index delivery and could be followed for another 3 years after the second delivery. The aim of this study was to determine the impact of GDM recurrence and pregnancy interval on glucose testing, primary care contact, and T2DM onset following the second delivery.

### Protection of human subjects

OLDW exposes data to researchers in certified, deidentified views. This study was therefore determined to be “not human research” by the Boston University Institutional Review Board.

### Variable construction

Index delivery represents the first livebirth delivery to an individual women recorded in this data set, but not necessarily the first livebirth to that woman. First delivery is the first mention in the dataset; gravidity and parity were not available. Glucose testing included any evidence for fasting blood sugar, hemoglobin A1c, or oral glucose tolerance test. Addition of hypoglycemic medication to nutrition and exercise counseling during the index pregnancy was used as a marker for GDM severity. Date of conception was estimated as 280 days before birth of a term, liveborn infant. Outcome variables include: glucose testing and primary care contact (claims evidence of a visit for any type of problem between deliveries), GDM in the subsequent pregnancy (with no evidence of T2DM onset before the third trimester), and onset of T2DM (new diagnosis code) by year-3 after the second delivery. OB/GYN specialists were not included in the definition of primary care, because long-term management of diabetes is not their expertise, and the recommendations for primary care follow-up from American Congress of Obstetricians and Gynecologists (ACOG) and American Diabetes Association (ADA) suggest the importance of referral for continued care. We looked at primary care visits for any type of problem, because contact of any type represented opportunity to monitor post-GDM. Variable specification is described in greater detail in the primary study from which this secondary analysis was drawn.^6^

### Analytic measures

We aggregated intervals from index delivery to subsequent conception into less than 1 year, 1–2 years, and then 2+ to 3 years postdelivery. We then conducted descriptive analyses to characterize the sample, identify the proportion of women who received glucose testing and primary care contact in the 3-year follow-up period after the index delivery, and determine the prevalence of GDM at the time of the second delivery. We used unadjusted and adjusted multivariable logistic regression analyses to assess the impact of GDM recurrence (noted at any time during the third trimester of the second delivery) and/or pregnancy interval (time from the index delivery to a conception that resulted in a live birth) on diagnosis of T2DM within 3 years after a second delivery. Variables entered into this analysis included those from the original study that were shown to be significant predictors of T2DM onset.

## Results

In this OLDW data set of 12,622 women with GDM in an index pregnancy, we found 1091 women who had a conception that resulted in a subsequent delivery within 3 years of the index delivery. Table 1 summarizes their demographic and medical characteristics, interval from delivery to next conception, and GDM recurrence noted by the time of the second delivery. We aggregated intervals from index delivery to subsequent conception as <1 year (*n* = 331), 1–2 years (*n* = 459), and >2–3 years postdelivery (*n* = 301).

### GDM recurrence, follow-up testing, and contact with primary care

Among women who conceived within 1 year of their index GDM delivery, 46.2% were GDM+ (*n* = 153), compared with 52.3% among women who conceived within 1–2 years (*n* = 240), and 48.2% among those who conceived 2+ to 3 years (*n* = 145), *p* = 0.22. Pregnancy interval (index GDM delivery to conception of the second pregnancy with a live birth delivery) was thus not associated with GDM recurrence (Table 2).

**Table 2.** Subsequent Pregnancy after Gestational Diabetes: Rates of Glucose Testing and Primary Care Contact by Gestational Diabetes Recurrence and by Pregnancy Interval

	GDM status	N	Glucose test within 3 years of subsequent delivery^a^ (%)	Primary care visit (%)
All continuously insured women with GDM affected livebirth, 2006–2012, followed through 2015		12,622	51	40
Exclusions				
No repeat pregnancy with delivery		10,432		
T2DM onset before repeat delivery		327		
Insufficient time for 3-year follow-up		772		
Second conception with term livebirth within 3 years (*n* = 1091)	GDM+	538 (49.3%)	53^*^	48
	GDM−	553 (50.7%)	31	45
Glucose testing and primary care contact by interval from delivery to next conception—conceived within				
1 Year after index delivery (*n* = 331)	GDM+	153 (46.2%)	48^*^	40
	GDM−	178 (53.8%)	31	49
1–2 Years after index delivery (*n* = 459)	GDM+	240 (52.3%)	53^*^	48
	GDM−	219 (47.7%)	35	42
>2–3 Years after index delivery (*n* = 301)	GDM+	145 (48.2%)	58^*^	55
	GDM−	156 (51.8%)	26	46

^a^ Any FBS, HbA1c, or OGTT within 3 years after a second livebirth delivery, excluding any tests associated with a GDM or T2DM claim, or tests that occurred before the second conception or during a subsequent pregnancy.

^*^ *p* < 0.001.

FBS, fasting blood sugar; HbA1c, hemoglobin A1c; OGTT, oral glucose tolerance test; T2DM, type 2 diabetes.

Fewer than half of the women in the parent study received postpartum glucose testing within the recommended postdelivery interval after their initial GDM-associated delivery.^6^ Those with recurrent GDM were significantly more likely to receive any form of glucose testing after their second delivery compared with women who did not experience recurrence in a subsequent pregnancy (53% vs. 31%, *p* = 0.001), and this pattern held across all three pregnancy interval groups. Rates of primary care contact were low overall (46%) and did not differ by GDM recurrence or interval to next conception.

### Impact of interval to next conception on T2DM onset

A shorter interval between a GDM affected pregnancy and next conception increased the likelihood of developing T2DM within the 3 years after the second delivery (*p* = 0.03). Among women with a second pregnancy conceived within a 1 year interval (*n* = 331), 25 (7.6%) experienced T2DM onset in the follow-up period. Among those with a second pregnancy conceived 1+ through 3 years after GDM (*n* = 760), 33 women (4.3%) developed T2DM (*p* = 0.03).

### Impact of GDM recurrence on T2DM onset

The 538 women with GDM recurrence had a higher proportion with T2DM onset within 3 years than the 553 women with no recurrence (*n* = 44 or 8% vs. *n* = 14 or 3%, *p* = 0.001). Although pregnancy interval was significantly associated with onset for the group as a whole as described above, within the subgroup of women with GDM recurrence, pregnancy interval did not have a statistically significant impact on T2DM onset (Table 2).

### Factors predicting T2DM onset

In unadjusted analyses a shorter pregnancy interval (delivery <1 year after the index delivery, compared with >2 years) increased the odds of T2DM onset within 3 years after the second delivery (odds ratio [OR] 2.38, 95% confidence interval [CI] 1.12–5.04). In adjusted analyses, GDM severity in the index pregnancy was a strong independent predictor of subsequent T2DM onset (OR 2.36, 95% CI 1.31–4.27), as was Hispanic race (OR 2.13, 95% CI 1.04–4.35). GDM recurrence nearly tripled the odds of T2DM onset (OR 2.92, 95% CI 1.54–5.53). When we adjusted for age, race/ethnicity, educational level, GDM severity, and excess gestational weight gain in the index pregnancy, the effect of a short pregnancy interval on the odds of developing T2DM was attenuated to a trend (OR 1.99, 95% CI 0.92–4.33, *p* = 0.08) (Table 3).

**Table 3.** Predictors of Type 2 Diabetes Onset After Gestational Diabetes: Adjusted Analysis

Variable	Unadjusted OR	95% CI	Adjusted OR	95% CI
Age 35+ vs. <35			1.64	0.92–2.94
Race: Asian vs. white			0.92	0.35–2.44
Black vs. white			1.63	0.60–4.45
Hispanic vs. white			2.13	1.04–4.35^*^
Some college/degree vs. less			0.58	0.33–1.01
Excess gestational weight gain			1.08	0.25–4.71
GDM therapy: medication required			2.36	1.31–4.27^*^
GDM recurrence in second pregnancy			2.92	1.54–5.53^*^
Timing of second conception with livebirth^a^				
1–2 years vs. 2–3 years after index GDM delivery	1.53	0.72–3.27	1.41	0.65–3.06
≤1 year vs. 2–3 years after index GDM delivery	2.38	1.12–5.04^**^	2.00	0.92–4.33^***^

^a^ Small cell size for several predictor variables resulted in suppression because of deidentification restrictions within OLDW; as a result we are unable to report on an interval of <1 year versus 1–2 years.

^*^ *p* < 0.01; ^**^p < 0.03; ^***^p = 0.08.

CI, confidence interval; OLDW, OptumLabs Data Warehouse; OR, odds ratio.

## Discussion

There is an abundance of literature about rates of GDM recurrence and T2DM onset, but little has been published about the contribution of the interpregnancy interval to either the risk of GDM recurrence or to postdelivery onset of T2DM. This study investigated the impact of GDM recurrence and interval to next conception as predictors of T2DM onset after an initial GDM-affected delivery. Two of our key findings have important clinical implications. First, we were able to confirm a recurrence risk for GDM of ∼50% for subsequent pregnancies, regardless of interval between deliveries. Second, for the group as a whole, a short interval between the initial GDM-affected delivery and subsequent pregnancy resulting in a term livebirth increased the likelihood of early onset T2DM. In a multivariate analysis that included both GDM recurrence and initial GDM severity, a pregnancy interval of less than a year increased the risk of T2DM onset within 3 years following the second delivery, compared with a 1–2-year interval (OR 2.00, 95% CI 0.92–4.33, *p* < 0.08). This trend suggests that the risk of T2DM may be affected by close pregnancies. This result calls for further investigation in a larger sample. In addition, these are continuously insured women, so the magnitude of the risk is likely even higher among underserved minority women who bear a greater burden of glucose intolerance and substantial barriers to care. Our preliminary finding of a possible association between time to next pregnancy and T2DM onset suggests the importance of contraceptive counseling after a GDM-affected delivery.

The strength of this study is the analysis of the impact of repeat pregnancy interval in a large data set of continuously commercially insured women with an initial GDM-affected delivery. In general, studies of GDM are small and heterogeneous in terms of socioeconomic demographics. Continuous insurance coverage represents an analysis of follow-up and outcomes holding financial access constant because lack of insurance does not present barriers to seeking care. Because a high number of women in the general population do not follow-up a GDM pregnancy within 5 years to allow for emerging changes in glucose metabolism to be identified,^7,8^ some with aberrant glucose regulation go undetected, and the real risk of early onset of T2DM is likely underrepresented. Continuous insurance enrollment (uninterrupted access) thus limits the potential for confounding of data for T2DM onset. In even this continuously insured sample initially reported, a third of the women lacked follow-up^6^ as stipulated by established glucose testing and primary care referral guidelines from the ACOG and the ADA.^8–10^ Because of this potential for undiagnosed cases, the rate of early onset T2DM may actually be higher than even our data from continuously insured women would suggest; future longitudinal studies to assess the full 10-year span in which T2DM onset is most likely to occur would clarify this issue.

The finding of discordance between GDM recurrence rates and T2DM onset as related to interpregnancy interval raises a possibility that although these two entities have much in common, their biological paths may be different. Since this is an observational study, we do not have a basis for suggesting a hypothesis for difference in biology.

In general clinical practice there has been great emphasis on measures to enhance access to long-acting reversible contraceptives (LARCS) to increase the interval between pregnancies and improve pregnancy and delivery outcomes.^11^ We undertook this study of the impact of delivery interval on recurrence and T2DM onset to see if enhancing contraceptive use and effectiveness might be a pathway to reducing the occurrence of T2DM after a GDM-affected pregnancy. There are several accessible points after delivery to counsel about interpregnancy interval and dispense effective contraception, highlighting the importance of pregnancy spacing on women's health. Obstetricians can use the postpartum encounter very appropriately to arrange for immediate postpartum glucose testing, recommend a preventive nutrition and exercise program, discuss the value of transfer to primary care for continued follow-up, and address the potential benefits of a longer time to next conception. Immediate postplacental or postpartum placement of LARCS is especially effective in increasing the interpregnancy interval and in turn possibly reducing the risk of T2DM.^12^ A discussion at the postpartum visit of the potential benefits of prevention of diabetes and chronic illness and the variety of methods available for contraception may be compelling enough to increase acceptance and address an important, currently neglected, component among efforts to curb the diabetes epidemic. Our results indicate that clinicians, researchers, health care innovators, and policy makers should still concentrate on efforts to reduce known barriers to postpartum glucose testing and primary care access,^13^ and consider adding contraception use and effectiveness to this effort.

### Limitations

The limitations of this study include the lack of information from the electronic medical record about interpregnancy weight gain, an independent risk factor for developing T2DM. Increasing body mass index (BMI) between pregnancies is known to add to the risk of GDM in a subsequent pregnancy, more so for women who are normal weight than for those who already have an elevated BMI.^14^ In a retrospective cohort study of 1401 nulliparous patients with GDM followed for up to 13 years, a subsequent pregnancy complicated by GDM was associated with a relative risk of 2.3 (CI 1.6–3.4) of developing T2DM. In this same study any weight gain was associated with an increased risk of developing T2DM (risk ratio 2.6, CI 1.2–5.5) for women who gained 5.1–10 kg.^15^ While we did not have BMI available for comparisons, we did control for excess gestational weight gain during either pregnancy (using the ICD-9 diagnostic code), and found no significant contribution of this code to T2DM onset within the 3-year follow-up period.^6^ Gravidity and parity may be predictive factors, but are not available in any large administrative data set; we chose to study the larger numbers available to us through “big data” because clinical data sets, while richer, do not offer an adequate sample size for a longitudinal analysis, but we may have missed women who had GDM before enrollment in this data set.

Furthermore, the sample was intentionally restricted to continuously insured women. This exclusion gave us power to evaluate key predictors of follow-up beyond major financial barriers. The loss of ∼80,000 women with GDM (8.2% of the 1,004,376 who were excluded for episodic insurance) may have limited generalizability. In this trade-off, the study gained internal validity at the cost of external validity. While this sample does not purport to be representative of all U.S. women, the prevalence of GDM among the women we studied was comparable to the national average, comparability studies were similar for key factors, and the clinical profile as reported previously does not differ markedly from national reports of indicators related to GDM prevalence and pregnancy and delivery complications.^6^ Follow-up glucose testing rates were also similar to those found in other types of samples.^7,8^

## Conclusions

Onset of T2DM after GDM can be prevented or mitigated, but many women do not receive recommended follow-up monitoring and preventive care. GDM recurrence tripled the odds of early T2DM onset within a 3-year follow-up period after the second delivery. Less time to next conception resulting in a livebirth was associated with an increased likelihood of T2DM onset, although the trend did not reach significance, in part because of the strength of GDM recurrence as a predictor of T2DM onset. Follow-up study is needed, but our findings suggest that an interval of a year or more between a GDM delivery and the next conception resulting in a livebirth may be beneficial for diabetes prevention. The postpartum visit is an ideal time to go beyond wound checks and usual issues to inform patients about T2DM risk and prevention opportunities, and discuss optimal spacing of future pregnancies.

## Abbreviations Used

ACOG: American Congress of Obstetricians and Gynecologists
ADA: American Diabetes Association
CI: 95% confidence interval
FBS: fasting blood sugar
GDM: gestational diabetes
HbA1c: hemoglobin A1c
ICD-9: International Classification of Diseases, 9th revision
LARCS: long-acting reversible contraceptives
OGTT: oral glucose tolerance test
OLDW: OptumLabs Data Warehouse
OR: odds ratio
T2DM: type 2 diabetes
